# Performance Assessment of Distributed Strain Sensing Techniques for Convergence Monitoring of Radioactive Waste Repository

**DOI:** 10.3390/s23010398

**Published:** 2022-12-30

**Authors:** Arianna Piccolo, Yann Lecieux, Sylvie Lesoille, Pierre Teixeira, Johan Bertrand, Dominique Leduc

**Affiliations:** 1 French National Radioactive Waste Management Agency (Andra), 92298 Chatenay-Malabry, France; 2 Laboratoire GeM UMR 6183, Nantes Université, 44000 Nantes, France; 3 Département Instrumentation d’EGIS, 38180 Seyssins, France

**Keywords:** Structural Health Monitoring, distributed optical fiber, strain sensing cable, Rayleigh scattering, Brillouin scattering, convergence monitoring, shape monitoring

## Abstract

This paper presents the measurement methodology of diameter reduction monitoring of micro-tunnel structures used for radioactive waste storage based on distributed strain measurements along fiber optic sensors installed on the circumference. The whole measurement procedure is described: the calibration of the sensors for use in harsh environment (temperature and radioactivity), the measurement analysis technique, the performance assessment of different measurement systems on a surface mock-up and the in-situ validation on an underground structure. The performances of Brillouin and Rayleigh backscattering measurements are compared, as well as different fixation technologies. Distributed measurements are compared to alternative measurements: displacement sensors, Bragg grating extensometers and MEMS accelerometers. The distributed Rayleigh backscattering measurement performed on optical cables bonded to the surface of the structure appears to be the best solution for monitoring the convergence of micro-tunnels and offers comparable performance to alternative technologies tested on the surface demonstrator.

## 1. Introduction

The French National Agency for Radioactive Waste Management (Andra) has been commissioned, under the French law, to build an underground repository for long-lived radioactive waste, those that cannot be disposed in surface. The underground center, called Cigéo, will be built at around 500 m depth underground in the Callovo-Oxfordian clay layer [1]. As by law the project must be reversible for the duration of the secular operating period, the waste must be in turn retrievable. Andra is currently evaluating methods for monitoring disposal cells in its Underground Research Laboratory, where sensors of different types are operated on waste disposal cell demonstrators [2]. These cells are similar to real disposal cells and placed in galleries dug in the same Callovo-Oxfordian clay layer and at the same depth as the one in which Cigéo will be built.

This paper focuses on the use of distributed measurements [3,4] with optical fibers for the diameter variation monitoring, also called convergence monitoring, of cells that will contain high level waste. Disposal packages will be inserted in subhorizontal dead-end tunnels, from 80 m to 150 m length, constructed and operated from the access shafts. Each tunnel has an inner lining consisting of a 25 mm-thick non-allow steel tube of 600–700 mm effective diameter, subjected to mechanical loading from the rocks.

Convergence monitoring is usually done with measurements instruments, such as 3D geodetics total stations, inclinometers or laser telemeters, fixed on the tunnel vault [5,6,7,8,9,10,11,12]. This kind of instrumentation is well suited for railway or road tunnels where sufficient space is available, but is not possible for waste repository in which waste left no place to cumbersome sensors. The alternative that first springs to mind is to use optical fiber sensors and to perform distributed measurements in order to get the maximum of information. This also allows for remote measurements in which all active components (lasers and detectors) are out of the harmful zones.

Distributed optical fiber sensing is based on the scattering processes that take place within the fiber. Microscopic or macroscopic variations in density, composition or structure of the material cause Rayleigh scattering. This is a linear and elastic scattering that creates a backward-propagating wave. Sound waves or acoustic phonons give rise to the so-called Brillouin scattering, a non-linear and inelastic effect where the scatter occurs at some GHz of frequency shift. The major advantage of scattering-based measures is that each section (of length equal to the resolution) of the fiber is a sensor which allows to obtain the value of the measured quantity all along the fiber. In the case of Brillouin scattering, this value is directly a frequency shift. For Rayleigh scattering, the fiber was interrogated with the Tunable Wavelength Coherent Optical Time Domain Reflectometry (TW-COTDR) method which takes advantage of the coherent configuration to obtain results by the cross-correlation of the Rayleigh scattering “fingerprints” traces of the sensors [13]. The measured quantity with this method is also a frequency shit. For both Brillouin and Rayleigh scattering, it can the be written:

(1) $$\Delta \nu =C_{\varepsilon }\Delta \varepsilon +C_{T}\Delta T$$

where $C_{\varepsilon }$ is the sensitivity to strain and $C_{T}$ is the sensitivity to temperature. Numerous works have been made on the use of distributed optical fiber sensing for the monitoring of tunnel (see [14,15] and references in there), but these works focused on strain measurement, and none dealt the problem of the transition from strain measurement to convergence, as it is done here.

The objective of this article is to present in a synthetic way and in one place the complete methodology to perform shape monitoring of tunnel like structure in radiactive environment. The different stages are presented in detail as follows: the qualification of the optical fibers and the cables in harsh environment, the development of the measurements analysis method, its qualification in laboratory conditions and finally the in-situ validation of the whole process. Some known results will be presented in a new light and particular attention will be paid to new elements, especially the comparison between the measurements made using Rayleigh or Brillouin scattering, the anchoring technologies and to the comparison with measurements made with alternative devices. A synthetic feedback of the methods and technologies used by Andra to monitor disposal cells is provided. It could serve as a guideline for the instrumentation of similar structures.

## 2. Optical Fibers in Harsh Environment

The environment of the targeted application combines radiations with a 1 MGy dose expected after 100 years and heat with a temperature around 80 °C. Radiation degrades the optical fiber properties through three different mechanisms [16,17]: radiation-induced attenuation (RIA), radiation-induced emission (RIE) and compaction. The RIA increases the linear attenuation and as a consequence reduces the distance range for both Rayleigh and Brillouin measurements. Compaction leads to changes in the density of silica. This corresponds to a change in the refractive index and, consequently, a change in the frequency shift obtained from backscattering measurements. The RIA is lower for fluor-doped fiber than for germanium-doped ones, as well as the radiation induced frequency shift [18].

When temperature and radiation are considered together [19], it can be observed a shift in Brillouin frequency of 3 MHz for Ge-doped fiber and 2 MHz for Fe-doped fiber, for a dose of 1 MGy whatever the temperature. These shifts correspond respectively to an apparent strain of 60 με and 40 με. The frequency shift for Rayleigh scattering is 6 GHz for both fibers. This corresponds to an apparent strain of 40 με. The expected strain after 100 years is at least of the order of 1000 με. It will then be possible to use optical fiber in radiation environment, as the radiation impact is almost negligible overall for strain sensing purposes.

The radiation induced Brillouin gain attenuation and the RIA are lowered by temperature. For a dose of 1 MGy, the measurement distance range is increased from 50 m at 20 °C to 120 m at 80–100 °C for Ge-doped fibers, and from 200 m to 500 m for Fe-doped fibers. As a consequence, the ageing qualification of optical fiber sensing systems must be performed taking into account temperature and radiation altogether.

## 3. Optical Cables in Harsh Environment

For a use in harsh environment, optical fibers are inserted inside cables made of several layers of different polymers and often a metallic layer to strengthen the cable. This outer coating protects the fiber against mechanical damage and acts as a barrier to lateral forces and moisture [20,21]. The cable considered here is a BRUsens V9 cable made by Solifos AG [22]. It is a 3.2 mm diameter shielded cable comprising an optical fiber with its primary coating surrounded by a multilayers buffer, a metal tube and then a structured polyamide outer sheath. For a better understanding, the cable and its constituent parts have been tested. In the following, V9 stands for a whole cable, FIMT for a fiber in metal tube and FO for a fiber with its primary coating only. Two kinds of fiber have been used: classical bending-loss insensitive G657 single mode fibers and fluor doped fiber with a special carbon coating [23] preventing hydrogen emitted by radioactive waste or by radiolyse from entering the optical fiber and obscuring it [24]. The samples with the latter fibers are marked with an subscript “F”. Irradiated samples are marked with a symbol 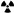 in exponent.

The measurement of the sensitivity of optical cables to strain is made by subjecting them to tensile tests performed by fixing the samples on a 10 m manual traction bench. The cables are stretched with a winch, while the actual length under test is measured with a millimeter-accurate laser telemeter. The measurements are acquired at each elongation step of 500 με in tension (i.e., a nominal value of 5 mm of elongation). Every 1000 με, the sample is returned to the initial position to measure any residual strain. Once the maximum strain threshold specified in the Cigéo project specifications has been reached (i.e., 10,000 με), the measurements are taken in increments of 1000 με, without returning the sample to its initial position, until a strain of 20,000 με or until the point of failure.

The optical cables are also subjected to thermal cycles to obtain their sensitivity to temperature and in order to understand the impact of a thermal cycle on the sensitivity of the sensors to the measurement of strain. From room temperature (20 °C), the samples are heated to the maximum temperature of 120 °C, slightly above the maximum temperature expected during the Cigéo monitoring phase. They are then cooled to −20 °C, then reheated to room temperature. The temperature changes are made by steps of 10 °C. For each step, the samples are kept at the same temperature for 60 min, in order to ensure the homogeneity of the temperature in the core of the optical fiber.

The frequency shift versus strain curves exhibit a break in slope around 2000 με (0.2%) which corresponds to the plasticization threshold of steel. Two different linear least square fittings were then performed for each cable, one for ε<2000με and one for ε>2000με. The slope of a given fitted line is the sensitivity to strain $C_{\varepsilon }$. As recommended by the BIPM [25] the uncertainty on this slope is given by:

(2) $$s\left(C_{\varepsilon }\right)=\sqrt{N\frac{s^{2}\left(\mathrm{residuals}\right)}{s^{2}\left(\varepsilon \right)}}$$

where *N* is the number of measurements, $s^{2}\left(\mathrm{residuals}\right)$ is the variance of the difference between the measured frequency shifts and the values given by the linear fit and $s^{2}\left(\varepsilon \right)$ is the variance of the measured strains. For each cable, the relative uncertainty on the sensitivity to strain is of the order of 1%.

**Figure 1:**
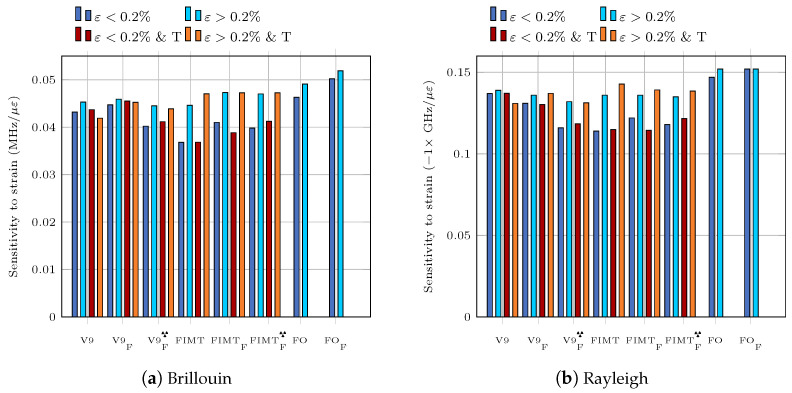
Sensitivity to strain of different cables.

Figure 1 shows that the strain sensitivity coefficient is systematically higher for ε>2000με due to possible plasticization and a modification of the cable’s constituent layers under traction. It is the FIMT type structure for which the sensitivity is the most affected. In the case of the V9 cables, the sensitivity to strain before and after 2000 με differs by less than 4% for the Brillouin measurements. This variation is less for the V9${}_{F}$ cable. It reaches nevertheless 10% for the case of Rayleigh measurements. The strain history undergone by the structure must be considered to avoid erroneous interpretations.

The exposure of the cables to radiations results in a decrease in sensitivity. It is probably related to the opacification of the optical fiber. The variation in sensitivity between the 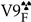 and V9${}_{F}$ cables is of the order of 10% for the Brillouin measurement and of 11% for the Rayleigh measurement. Without being prohibitive, this variation shows that a correction of the sensitivity coefficients should be considered over time in the context of long-term monitoring of elements subjected to radioactive radiation.

More generally, the mechanical tests show that radiation appears to have an impact on the outer polyamide sheath of the cable, reducing its ductility and leading to early cracking and failure.

**Figure 2:**
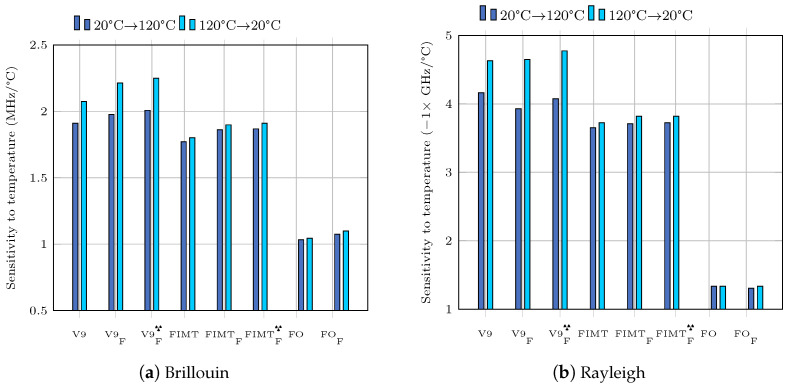
Sensitivity to temperature of different cables.

Linear least square fittings were also used to determine sensitivities to temperature. The relative uncertainties, calculated with Equation (2), are of the order of 0.2%. Figure 2 shows that the sensitivity of the Brillouin or Rayleigh frequency shift to temperature is greater for cable than for FIMT or fiber. This result is logical in the sense that the cable is a multi-layer structure of glass, steel and plastic. The outer plastic layer has an order of magnitude higher coefficient of thermal expansion than steel or glass. For cable-type structures, the hysteresis of the response to thermal loading induces a change in sensitivity. This effect is more pronounced under the effect of radiation. On the other hand, for a given thermal cycle, the values of the temperature sensitivity coefficients are very close to each other between irradiated and non-irradiated samples, which is an important point in the context of radioactive elements monitoring.

As a conclusion, the combination of strain above 2000 με, temperature cycling, and radiation changes the strain and temperature sensitivity coefficients of the optical cables by up to about 10% of the original values. It is therefore reasonable to consider this 10% factor as a measurement uncertainty.

## 4. Analysis Method

**Figure 3:**
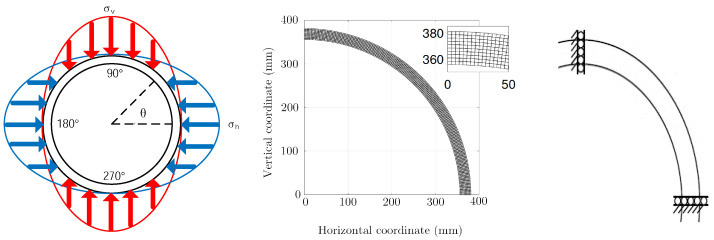
Modeling of static loading along the main horizontal and vertical stress directions $\sigma_{h}$ and $\sigma_{v}$ imposed on the HLW cell model. The structure being symmetrical, only the quarter is modeled with a mesh of 180 elements in the circumference and 10 elements in the thickness, the boundary conditions are also represented.

The behavior of the steel liner is assumed to be linear elastic and the model (geometry, boundary conditions, materials) is known. The external loads are modeled by two sinusoidal distributions in the horizontal and vertical directions: $\sigma_{h}$ and $\sigma_{v}$. This choice is guided by the fact that in the final device the loading is due to rock pressure [26]. The directions of loading are known. These are the eigenvectors of the stress tensor in the rock. This is a static loading of the “bearing” type. The loading model used for this simulation is shown in Figure 3 and is expressed as:

(3) $$\left\{\begin{array}{ccccc} \sigma_{h} & = & \alpha \sigma_{h}^{u} & = & \alpha \cos(\theta )\\ \sigma_{v} & = & \beta \sigma_{v}^{u} & = & \beta \sin(\theta ) \end{array}\right.$$

where α and β are the magnitudes of the stress to be calculated and θ is the angle around the structure. The finite element model allows to compute the unit strains $\varepsilon_{h}^{u}$ and $\varepsilon_{v}^{u}$ from the unit loads. Any strain ε(α,β) is then obtained by superimposing the unit strains:

(4) $$\varepsilon \left(\alpha ,\beta \right)\;=\;\alpha \varepsilon_{h}^{u}\;+\;\beta \varepsilon_{h}^{v}$$

The identification problem is then reformulated into a problem of minimization of a “cost function” which ensures the existence of a solution. The problem consists in finding the load parameters α,β which minimize the objective function Φ such that:

(5) $$\Phi \left(\alpha ,\beta \right)\;=\;∥\tilde{\varepsilon }{-\varepsilon \left(\alpha ,\beta \right)∥}_{2}\;$$

where $\tilde{\varepsilon }$ is the strain measured along the fiber and $\varepsilon \left(\alpha ,\beta \right)$ is the strain calculated by the finite element method.

The proposed model is deliberately chosen to be simple with few parameters. This is its main interest. Indeed, as in all identification problems, the uniqueness of the solution is not guaranteed. In order to reduce as much as possible the number of local minima of the cost function, it seems preferable on the one hand to use all the available experimental information and on the other hand to limit as much as possible the number of parameters to identify. Moreover, the finite element model allows to calculate the variation of the cell diameter at any point of the section.

## 5. Measurements on Mock-Up

### 5.1. Experimental Set-Up

**Figure 4:**
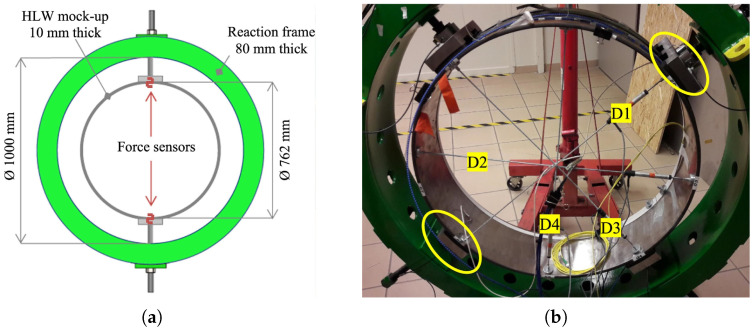
Test mock-up. (**a**) Scheme of the mock-up. (**b**) Full experimental setup: reaction frame (in green) with instrumented metallic ring. In detail the four displacement sensors “D#” and the load application runners (yellow encircled).

The method was first tested on a mock-up [27] for validation in collaboration with Egis Géotechnique (Grenoble, FR). The test device is a steel ring of 762 mm outer diameter, 10 mm thickness and 200 mm width. The steel has a Young’s modulus of 210,000 N/mm${}^{2}$ and a Poisson’s ratio of 0.28. A change in diameter from 0 to 10 mm is possible along two orthogonal directions. It is achieved by loading the ring on a reaction piece (in green in the Figure 4) with a pad attached to a rod screwed into the support piece.

**Figure 5:**
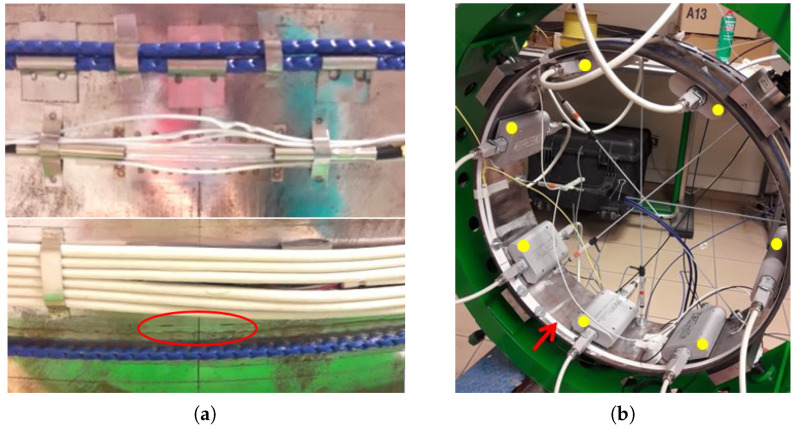
Test mock-up: positions of sensors. (**a**) From top to bottom: soldered cable, soldered FBG, electrical cables for resistive sensors alimentation, glued FBG (circled in red) and glued cable. (**b**) Sensuron sensing cable (red arrow) and Morphosense sensors (yellow points).

The ring is equipped with several sensor types. 4 displacement sensors with a resolution of 0.01 mm, are placed inside the ring along 4 diameters (D1 to D4 on Figure 4). These sensors are used as reference since they are similar to those used for the monitoring of micro-tunnels radioactive waste repository. On the external face of the ring, V9 cables and fiber Bragg gratings are fixed. One turn of V9 cable is glued all along its length (with Araldite 2021-1 glue) and one is fixed with a spot welding technique, where soldered supports are distanced from each other of about 4 cm. Eight FBGs have been fixed welding the extremities on the external circumference of the mock-up, at specific angular positions. At the same time, a 250 μm fiber with inscribed four FBGs was glued on the extrados with the EPO-TEK 302 glue, while kept in light tension to be able to properly measure compression (the imposed strain was in the order of 1500 με). On the internal face of the ring, 8 Morphosense sensors are attached and a Sensuron optical cable is glued (Figure 5b).

A force sensor is mounted on each movable runner, which applies the load to the structure. Four of these sensors are employed, being of the FTCN series (sensors of traction/compression) from the company Mesurex with a maximum measurable load of 10 kN and a resolution of 0.1%.

### 5.2. Brillouin vs. Rayleigh

Before examining the performance of distributed measurements, it is instructive to look at measurements obtained by Brillouin and Rayleigh scattering to identify which measurement protocols are best for each scattering and in which case each scattering is most appropriate.

The cables are interrogated with the Neubrescope NBX-7020 by Neubrex, using both Brillouin (PPP-BOTDA) and Rayleigh (TW-COTDR) scatterings. The measurement parameters are summarized in Table 1.

The load is applied along one direction (vertical direction) only. In this case, the parameter α in Equation (4) is null and the optimization just runs on the parameter β.

**Figure 6:**
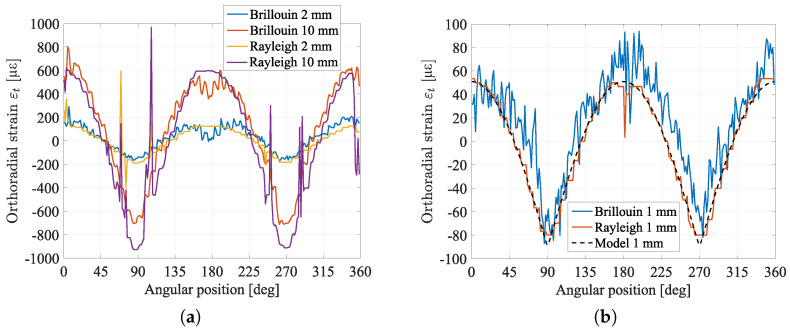
Comparison between Brillouin and Rayleigh scatterings. (**a**) Comparison for 2 mm and 10 mm of imposed displacement. (**b**) Comparison for low strain (1 mm) with the FE model simulation.

In Figure 6a Brillouin and Rayleigh scatterings strain measurements are reported for an imposed displacement of 2 mm and 10 mm, revealing a more regular and less noisy trace for Rayleigh than for Brillouin. More in detail, considering now the case where the convergence evolution is 1 mm, it is possible to quantify the measurement quality in the two cases by looking at the standard deviation of the traces which is defined as the mean over the angular position of the distance between measurement and model. The standard deviation of Brillouin measurement, equal to 17.3 με, is higher than the standard deviation of Rayleigh measurement, equal to 5.5 με, always in relation to the calculated model. For our strain sensitivity coefficients (C${}_{\varepsilon }^{B}$ = 0.0432 MHz/με and C${}_{\varepsilon }^{R}$ = −0.137 GHz/με), resolution is 0.75 GHz and 0.75 MHz for respectively Rayleigh and Brillouin scatterings. These values are in accordance with the acquisition parameters. It highlights that Rayleigh scattering is able to measure smaller strains. It is worthy to recall, however, that as Brillouin relies on central frequencies differences, it is easier to exploit than coherent Rayleigh scattering, which needs cross-correlation. Brillouin is more reliable and stable whichever the level of strain, while cross-correlation might fail if the frequency scan range is not sufficiently large.

**Figure 7:**
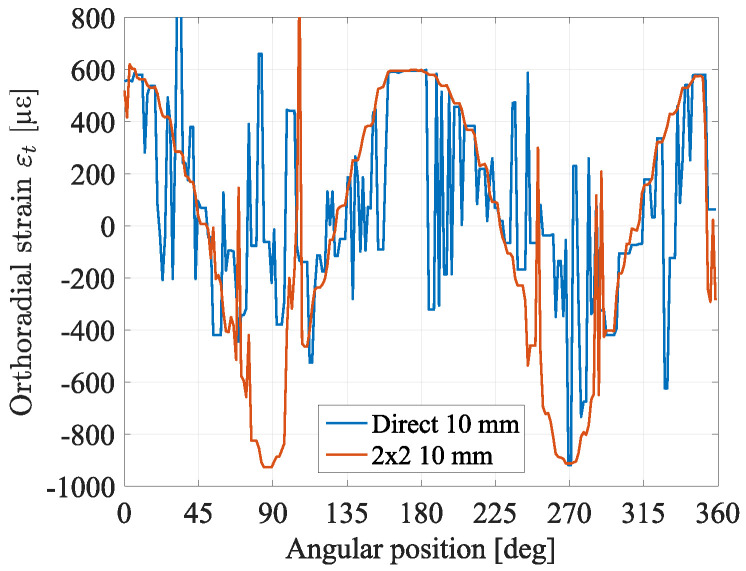
Comparison between two approaches of cross-correlation: “direct” is the direct cross-correlation between raw measurements and the reference, “2 × 2” is the cross-correlation between subsequent measurements.

In Figure 7 the comparison between the results of two cross-correlation approaches is shown, for an imposed convergence of 10 mm. Strain obtained by cross-correlating subsequent measurements (two-by-two, with step of 2 mm of convergence) is much more accurate than the one obtained via direct correlation with the reference (0 mm of convergence). Some error peaks are though still visible and this is due to the random nature of Rayleigh spectral response: they can occur in every cross-correlation based technique when strain levels reach several tens of microstrains. It was experimentally observed that the cross-correlation tends to fail when strain difference becomes higher than 500 με. As a first conclusion, it is preferable to cross-correlate subsequent measurements, two by two, in order to be sure that the difference between the two is minimal, summing then each trace up to the desired reference. This method is more time-consuming but more accurate than correlating each measurement directly to the selected reference. It also suggests that, for some applications when strain range is large, Brillouin scattering-based techniques are more robust.

### 5.3. Accuracy of Distributed Measurements Compared to Other Measurements

The displacement sensors are position transducers, pivot head mounting potentiometric up to 300 mm, of the Ingress Protection classification IP67 (IEC standard 60529) suitable for harsh environmental conditions, with a resolution better than 0.01 mm. The radial displacement is imposed at one extremity of D1, in order to measure exactly the desired convergence.

The Bragg sensors (FBG) are gratings inscribed in the core of the optical fiber. The length of the gratings is typically 1 cm which makes them localized sensors. Bragg gratings reflect a part of the incoming light centered on the Bragg Wavelength $\lambda_{B}$ which depends on the period of the grating and the effective refractive index of the fiber. When the grating is strained or submitted to a variation of temperature, its Bragg wavelength shifts:

(6) $$\Delta \lambda_{B}=C_{\varepsilon }\varepsilon +C_{T}\Delta T\;$$

The strain can then be retrieved from the measured Bragg wavelength shift if temperature is stable or known.

Brillouin or Rayleigh scattering measurements give a strain along the cable which can be transformed into orthoradial strain for the whole angular position on the cell circumference as shown in Figure 6 and Figure 7. Bragg gratings provide a local measurement of strain for fixed angular positions. These orthoradial measurements are converted into radial displacement using the method described in the Section 4.

For the Sensuron sensor, the software correlates strain measurement with curvature, retrieving the 3D shape of the instrumented structure. The sensor itself, already provided as part of the sensing system, is an optical fiber with inscribed quasi continuous FBGs, interrogated via an OFDR technique. The interrogator accuracy is given to be 1.25 με for strain and 0.15 °C for temperature. The calibration is done in two steps: one needs the cable to be straight, in the other it must be fixed on the structure. While the sensor is put in place it is possible to define and locate the measurement starting and ending points over the fiber length, in order to be able to obtain a curved and closed shape, taking as reference the straight cable calibration measurement.

**Figure 8:**
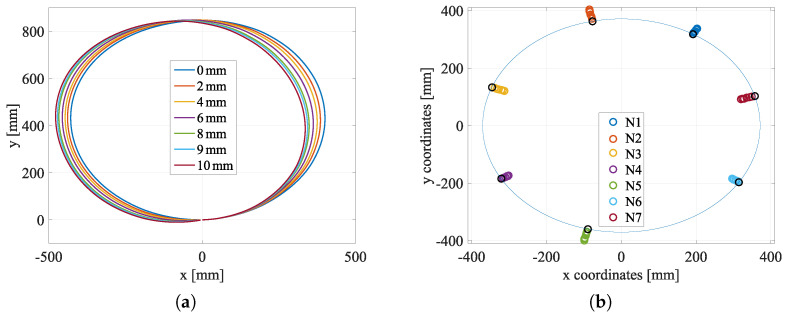
Measurements with Sensuron sensor and Morphosense sensors. (**a**) Sensuron 3D shape sensing: structure coordinates. (**b**) Morphosense convergence for each different MEMS sensor, with the original ring shape in a continuous blue line.

After the long calibration, measurements are very fast to acquire strain or 3D shape coordinates, as represented in Figure 8a for different imposed convergences. The shape of the structure is nicely observable. However, this figure shows an artifact related to the fact that rigid body movements are not detected. No method detects these movements but the origin of the axes for the other methods is at the center of the structure. For the Sensuron sensor, the zero of the coordinates is right where it was defined when implemented. This means that if the corresponding point on the structure moves due to the loading, it will not be detected with a measurement. To avoid the issue, the cable starting point has then to be chosen carefully and put, for example, where a displacement is imposed and therefore known. The raw measured values exhibit a bias due to the calibration of the strain measurement. In order to compensate this bias a coefficient 2.5 is systematically applied to measurements.

**Figure 9:**
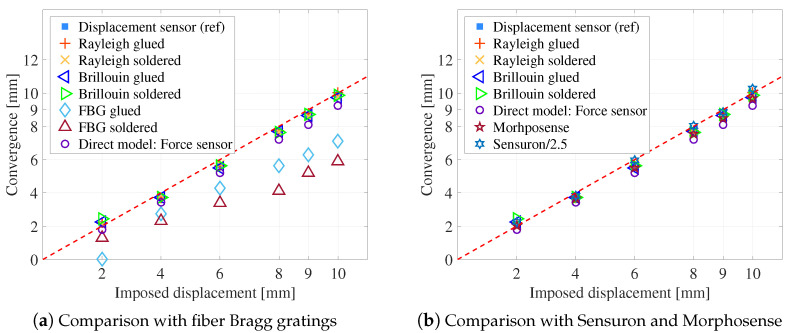
Convergence comparison between distributed optical fiber sensors and other systems.

The Figure 9 summarizes the convergences obtained with different measurement methods in the vertical direction. On these graphs are also plotted the convergences directly measured by the radial reference sensor and the convergence calculated with the finite element model using the force measured by the force sensor as input data. It appears that FBGs give the less accurate results. The error reaches 27% for glued FBGs and 40% for soldered FBGs. The difference between the two types of anchoring may be related to buckling in the compression parts despite pre-tensioning during installation. In any case, the low accuracy of these local measurements is due to the small number of measurement points and the high uncertainty in the angular localization of the sensors. Indeed, the strain curve presents steep portions (for example between 45° and 90° as can be seen on Figure 6) in which a small localization error results in a large convergence error.

**Figure 10:**
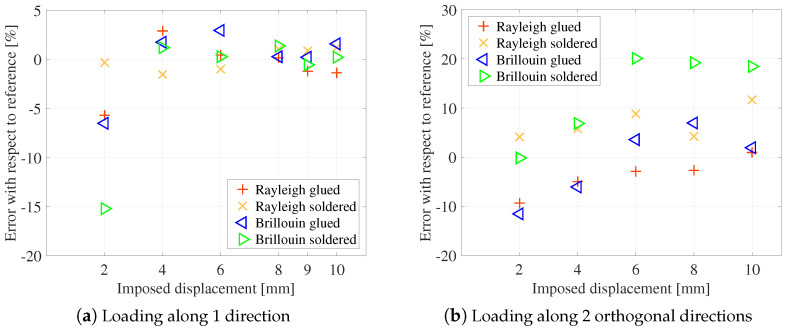
Error on convergence obtained by distributed measurement.

All the other measurement methods give consistent results within 1 mm of the reference value. The choice of one method or another will therefore be governed by considerations of ease of implementation or of calibration. For distributed measures, there is no clear indication of the best type of anchoring or the best type of diffusion. They are proving to be as effective as the established commercial measures. In particular, they allow a clear discrimination of the convergence steps, with an error lower than 5% for an imposed displacement higher than 2 mm (Figure 10a).

Experiments were also performed by imposing a loading in two orthogonal directions (vertical and horizontal). The optimization was then done on the two parameters α and β of Equation (4). Results are plotted on Figure 10b. Similar conclusions to the case of unidirectional loading can be drawn. However, it is observed in this case that soldered cables give less good results than glued cables. For the latter, the error on convergence is always less than 10%, which represents 1 mm for 10 mm of convergence. It may be related to cable buckling between two anchoring points.

These laboratory tests have confirmed the ability of the proposed method to retrieve the convergence from the strain.

## 6. In-Situ Measurements

**Figure 11:**
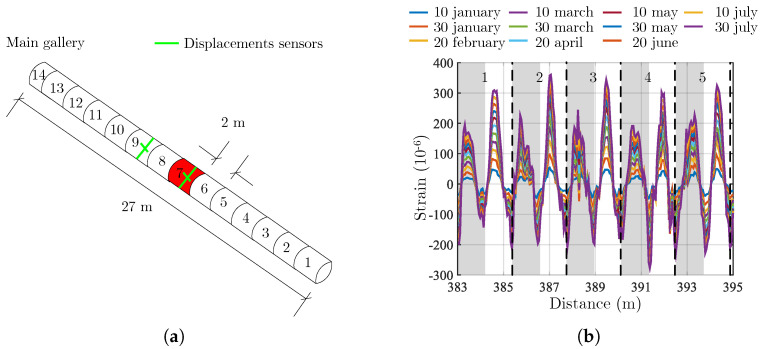
Scheme of the studied gallery and strain measurements. (**a**) Scheme of the studied gallery in the underground laboratory. The instrumented cell is in red and the displacement sensors are in green. (**b**) Strain measured along the fiber.

A scale one high level waste storage cell mock-up oriented along the principal stress directions was drilled in 2018 in the Underground Research Laboratory at the Meuse/Haute-Marne site. The outside diameter of the steel liner is 762 mm and the diameter of the gallery is 920 mm. The cell consists of 13 elements of 2 m length, of which the 7th is instrumented with a fiber optic cable that describes a helix of 5 turns for convergence monitoring [28]. Displacement sensors are also used to provide reference measurements. The feature under study is shown in red in Figure 11 and the displacement sensors in green.

The position of the zone of interest, where the cable is used to measure convergence, was located before inserting the metal liner into the rock. For this purpose discriminating points were heated to locate them along the optical path. The resulting strain can be divided into five parts (numbered 1 to 5 in the Figure 11b), each representing the same behavior. Each turn undergoes a sinusoidal strain related to the main stress directions. Compression in the vertical direction and elongation in the horizontal direction are observed resulting in a reduction of the vertical diameter. The strain measurements performed on the half-spires for which no obvious perturbation is observed are used as input data for the convergence calculation model. The measurements acquired on the half of the turns in gray are not used. Indeed, in these areas, the signal has peaks with very different values sometimes even for two consecutive points. This is the case for the signals recorded at a distance of 386 m and then at 388 m from the beginning of the fiber, i.e., on the gray area of towers 2 and 3. In zone 3, for example, two consecutive points for which the value of the strain is high and changes sign are visible. Such a variation cannot be the result of external loading. It is likely that these peaks are the result of a cross-correlation error of the Rayleigh signal. The cyclic nature of the position of the noisy zones suggests that there could be contact with the rock on one side of the coil and therefore of the cable, although this hypothesis cannot be verified.

**Figure 12:**
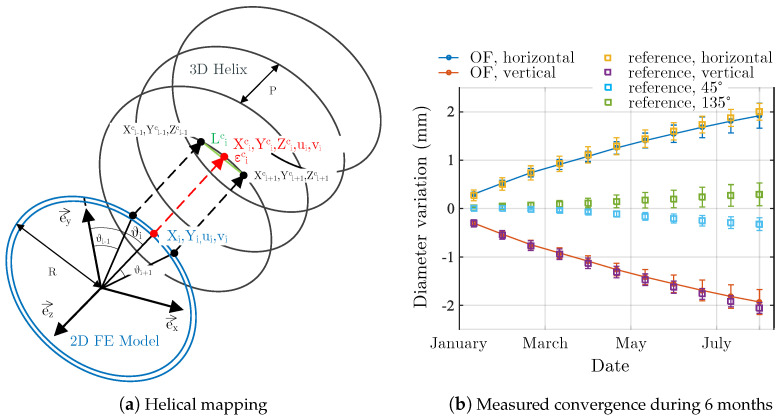
Mapping and first measurements.

The cell is considered as an infinite cylinder submitted to a radial loading given by Equation (3). Under these assumptions, the strain in any point of the cylinder can be calculates with a 2D model of the cross section. Firstly, the displacement $u_{i},v_{i}$ of the node $M_{i}\left(X_{i},Y_{i}\right)$ of the section are calculated using a the finite elements model. Then these displacements are mapped on the helix of pitch *P* corresponding to the optical fiber, $M\left(X_{i},Y_{i}\right)\to M^{c}\left(X_{i}^{c},Y_{i}^{c},Z_{i}^{c}\right)$ (see Figure 12) wich allows to convert the ortho-radial strain into a longitudinal strain along the fiber. This longitudinal strain is compared to the measured strain (Figure 11b). The optimization algorithm is implemented to minimize the fonction Φ(α,β) of Equation (5) and find the parameters α and β that give the best fit between the measured and calculated strains.

The Figure 12 shows the measurements of cell diameter variations during the first 6 months. The fiber optic measurement points are averages over different spires and the reference measurement points are averages over several displacement sensors. In both cases the uncertainty is about 0.3 mm. The difference between distributed and reference measurements starts at 20% when the diameter variations are small and falls to 10% when the variations reach 2 mm. This difference can be due to several causes:

- The strain transfer function of the cable is not exactly 1.
- The cells may have a little bit rotate during the installation. This is suggested by the variations measured by the reference sensors at 45° and 135° which should be null and are not. This little rotation induces an error on the directions of the loading.
- The installed optical fiber does not described a perfect helix, which induces an error on the mapping.
- The measurement points are localized with the hot point method with an uncertainty of 100 mm.
- The spatial resolution is lowered by the length of the structure. Only 20 measurement points per spire are available, which reduces the accuracy.

Despite these sources of uncertainty, it must be emphasized that the method allows to detect diameter variations of the order of 0.3 mm which meets the specifications of Cigéo monitoring program.

## 7. Conclusions

Based on the results presented in this article, it can be concluded that distributed strain sensing with Brillouin or Rayleigh scattering associated to a parametric identification calculation is an efficient method to monitor the convergence of tunnel:

- It can be used in harsh environment such as high level waste repository, in which the radiation dose is expected to be close to 1 MGy after 100 years and the temperature close to 80 °C. Under these conditions, the sensitivity to strain and the sensitivity to temperature vary by no more than 10%.
- It is well suitable for long structures since the measurement range with optical fibers can reach several kilometers.
- It allows to monitor the geometry of the structure at any point with a single cable.
- The inner space of the tunnel is unoccupied by sensors.
- The measurement analysis takes few minutes with an ordinary processor, this is fast enough to monitor in real time the structure.

It was also shown that Rayleigh scattering had a better resolution than Brillouin scattering, but that it should be used by correlating two nearby states. For large strain, Brillouin scattering gives better results. Furthermore, distributed measurements were found to be much better than localized Bragg grating measurements and comparable to measurements provided by commercially available reference sensors.

The advantage of distributed measurement is that it is possible to control very long optical fiber lengths. However, this capacity is at the expense of the acquisition frequency of the measurement on the one hand and its spatial resolution on the other hand. A compromise must therefore be found between these different parameters depending on the application, the desired information, the complexity of the parameterized model used for the analysis and the tolerated measurement uncertainty.

Concerning the practical implementation, the data analysis highlighted the difficulties inherent to this type of exercise. Obtaining consistent results requires methodical verification of the information provided by the client or the instrumentation team. The precise location of the fiber and its geometry (variable helix pitch) caused difficulties in interpreting the signals. This feedback shows that the health monitoring of structures requires an excellent knowledge of the real structure and its instrumentation (true geometry, boundary conditions and sensor position). An accurate record of this information and its archiving for future users is therefore essential.

Decorrelating the effects of temperature and strain is a tricky problem. In the case of the tests performed here, the temperature variations were negligible. In the case of the future application in radioactive waste repositories, the temperature is expected to be spatially uniform with a slow temporal evolution. A few Pt100 type temperature sensors could be sufficient to perform this decorrelation. An additional Raman-interrogated optical fiber could also be used. We are less confident in using combined Rayleigh and Brillouin scattering measurements because, as shown in Section 5.2, these methods have too different application domains to be combined effectively.

The proposed method is well adapted to galleries with a steel lining. For intermediate level long live waste concrete tunnel are selected. Its implementation on galleries with a concrete lining is expected to be more complex because of the effects of the evolution of the material at young age and the adhesion of the cable in the structure. In any case, the basis for the analysis is laid here and can be used as is.

## Figures and Tables

**Table 1:** Measurement parameters.

	Brillouin	Rayleigh
Spatial Resolution	2 cm	2 cm
Sampling Interval	1 cm	1 cm
Averaging Count	$2^{15}$	$2^{13}$
Probe Output Power	+1 dBm	–
Pump Output Power	+30 dBm	+26 dBm
Frequency Range	[10.45–10.88] GHz	[194–194.25] THz
Frequency Span1	MHz	500 MHz

## Data Availability

Data sharing not applicable.
